# Anterior Communicating Artery Aneurysms: Anatomical Considerations and Microsurgical Strategies

**DOI:** 10.3389/fneur.2020.01020

**Published:** 2020-09-08

**Authors:** Junhui Chen, Mingchang Li, Xun Zhu, Yan Chen, Chunlei Zhang, Wenwen Shi, Qianxue Chen, Yuhai Wang

**Affiliations:** ^1^Department of Neurosurgery, Renmin Hospital of Wuhan University, Wuhan, China; ^2^Department of Neurosurgery, 904th Hospital of Joint Logistic Support Force of PLA, Wuxi Clinical College of Anhui Medical University, Wuxi, China; ^3^Department of Internal Medicine, Hexian Hospital of Traditional Chinese Medicine, Ma'anshan, China

**Keywords:** AcoA aneurysm, anatomical, microsurgical strategies, clipping, coiling, surgical strategy

## Abstract

Anterior communicating artery aneurysms account for 23–40% of ruptured intracranial aneurysms and 12–15% of unruptured aneurysms and are the most common intracranial ruptured or unruptured aneurysms. Because they have relatively complex anatomical structures and anatomical variations and are adjacent to important blood vessels and structures, in the process of microsurgical exposure of an Anterior communicating artery aneurysm, attention should be paid not only to the anatomical characteristics of the aneurysm itself but also to the adjacent important blood vessels and perforating arteries; therefore, both surgical clipping and endovascular embolization are serious challenges for neurosurgeons. No matter which treatment is chosen, it is necessary to determine the structure of the Anterior communicating artery and its perforating arteries as well as whether there is a fenestration deformity of the Anterior communicating artery and the relationship between bilateral A1-A2 before surgery. The shape and size of the aneurysm itself and its location relative to adjacent blood vessels also need to be considered to better complete the procedure, and this is especially true for microsurgical clipping. Clarifying the anatomy before surgery is helpful for better selecting the surgical approach and surgical side, which could affect the intraoperative exposure of the aneurysm and adjacent arteries, the surgical difficulty, the resection rate, and the postoperative complications. Therefore, starting with Anterior communicating artery aneurysms and their adjacent structures and variations, this paper reviews the latest progress in surgical treatment based on anatomic specificity as well as the most recent clinical studies.

## A General Overview of the Anterior Communicating Artery (AcoA) Aneurysms

AcoA aneurysms are the most common intracranial aneurysms, accounting for 23–40% of intracranial aneurysms and 12–15% of unruptured aneurysms, and are the most common type of intracranial aneurysm in patients under 30 years old (1–3). AcoA aneurysms are more likely to rupture than are other types of intracranial aneurysms due to their anatomical and hemodynamic characteristics (3, 4). The international study of unruptured intracranial aneurysms (ISUIA) reports that smaller anterior circulation intracranial aneurysms are associated with lower risks of rupture and that the risk of rupture is <1% per year for anterior circulation aneurysms with a diameter <7 mm (2, 5–7). However, a large number of recent studies have found that AcoA aneurysms <7 mm still have a very high risk of rupture, accounting for ~40% of all ruptured intracranial aneurysms (6, 8). Bijlenga et al. (9) found that the risk of rupture risk for more than 900 cases of AcoA aneurysms with a size of 4–7 mm was similar to that for posterior circulation aneurysms. In a study of 200 cases of ruptured aneurysms, Lee et al. (10) found that 47% were small aneurysms (<5 mm) and that the most common rupture site was in the AcoA. The further in-depth study found that for microaneurysms (<3 mm), the rupture occurred more frequently in patients with hypertension combined with AcoA aneurysms. Therefore, for AcoA aneurysms, regardless of rupture and size, active intervention should be conducted once they are discovered.

At present, with the development of endovascular interventional techniques and interventional materials, increasingly more intracranial aneurysms can be embolized by endovascular techniques or treated with flow diverters; however, endovascular treatment cannot be performed due to the complex structure of AcoA aneurysms, such as a poor aspect ratio (dome/neck), inability to place catheters, and influencing blood flow in collateral blood vessels. Moon et al. (11) reviewed the long-term prognosis of AcoA aneurysms included in the Barrow Ruptured Aneurysm Trial (BRAT). After randomization, 91 patients (70%) with AcoA aneurysms were included in the clipping group, and 39 patients (30%) were included in were embolization group. In the embolization group, 16.9% of the patients crossed over from this group to the surgical clipping group due to embolization difficulties. No patients were transferred from surgical clipping to coiling embolization. There was no significant difference in clinical outcomes between the 2 groups after 1–3 years of follow-up (once per year). However, the retreatment rate for the surgical clipping group was superior to that for the embolization group. Among them, 3 patients (3.3%) have retreated in the clipping group, and 3 patients (7.7%) have retreated in the coiling embolization group.

Therefore, independent of intervention technology development, it is very difficult for endovascular treatment to solve all issues regarding AcoA aneurysms because of structural and hemodynamic complexities. Microsurgical clipping remains the safest and most basic treatment. However, AcoA aneurysms are different from other simple intracranial bifurcation aneurysms. AcoA aneurysms arise from a complex consisting of 5 arteries, including the A1 segment of the bilateral anterior cerebral artery, the A2 segment of the bilateral anterior cerebral artery, and the AcoA itself. Besides, another 7 arteries are adjacent to the AcoA complex, including the bilateral recurrent artery (recurrent artery of Heubner), the perforating branches of the AcoA, the bilateral orbitofrontal artery, and the bilateral frontopolar artery (7). The anatomical structure of the AcoA itself also involves the size and shape of the aneurysm: the aspect ratio (dome/neck), the direction and calcification of the aneurysm, and the presence or absence of a thrombosis inside the aneurysm. As a result, the anatomy of and surgery for AcoA aneurysms are varied and are very challenging. In this paper, we summarize the anatomy of and variations in AcoA aneurysms and review the latest advances in surgical treatment based on the specificity of its anatomy combined with clinical practice.

## Anatomical Characteristics of the Adjacent Structures of AcoA Aneurysms

AcoA aneurysms are relatively complicated for treatment with microsurgery because of their complex structure and adjacent vessels and tissues. The following is a brief description of important neighboring anatomical structures and variations, which helps understand surgical clipping strategies.

## The Main Trunk and Classification of the AcoA (Figure 1)

The AcoA is a short blood vessel that connects the bilateral anterior cerebral arteries. It is located above the optic chiasm and corresponds to the inferior portion of the lamina terminals. Kirgis et al. (12) and Yasargil (13) classified the AcoA into simple and complex according to the morphology of the blood vessels. For the former, the AcoA connects to the A1 segment of the bilateral anterior cerebral artery. Generally, ~40% of trunks are single, with a length of 1.5–8.8 mm (average, 4.0 mm) and a diameter of 0.2–2.5 mm (average, 1.7 mm). The complex type generally includes 2 branches, the “Y,” “H,” or “X” types, fenestration deformity, or the dimple or “O” type, accounting for ~60% of trunk types. Besides, in some individuals, the A1 segment of the bilateral anterior cerebral arteries is fused, exhibiting the absence of the AcoA, which accounts for approximately 4.5% of trunk types. The variation rate for the AcoA is extremely high, as high as 60%. The types of variation include plexiform (33%), dimple (33%), and fenestration deformity (21%) (14). It has been reported that the fenestration deformity of the AcoA is prone to misdiagnosis and mistreatment due to the current wide application of CT angiography (CTA). The slice thickness of CTA affects the spatial resolution, and image reconstruction and image softening cause an AcoA with fenestration deformity to be easily misdiagnosed as an aneurysm (15, 16). Therefore, when considering an AcoA aneurysm, the possibility of AcoA variations should be considered carefully, or digital subtraction angiography (47) should be performed to confirm the diagnosis and reduce the possibility of misdiagnosis. In terms of the microsurgical clipping of AcoA aneurysms, adequate preoperative assessment of the anatomical structure of the AcoA, especially the compensatory role of the AcoA for blood flow to the bilateral anterior cerebral artery, should be performed. Adequate preoperative assessments of the location of the AcoA aneurysm and its relationship with AcoA variations are extremely important for ensuring successful completion of the surgery and the safety of the operation (17, 18).

**Figure 1:**
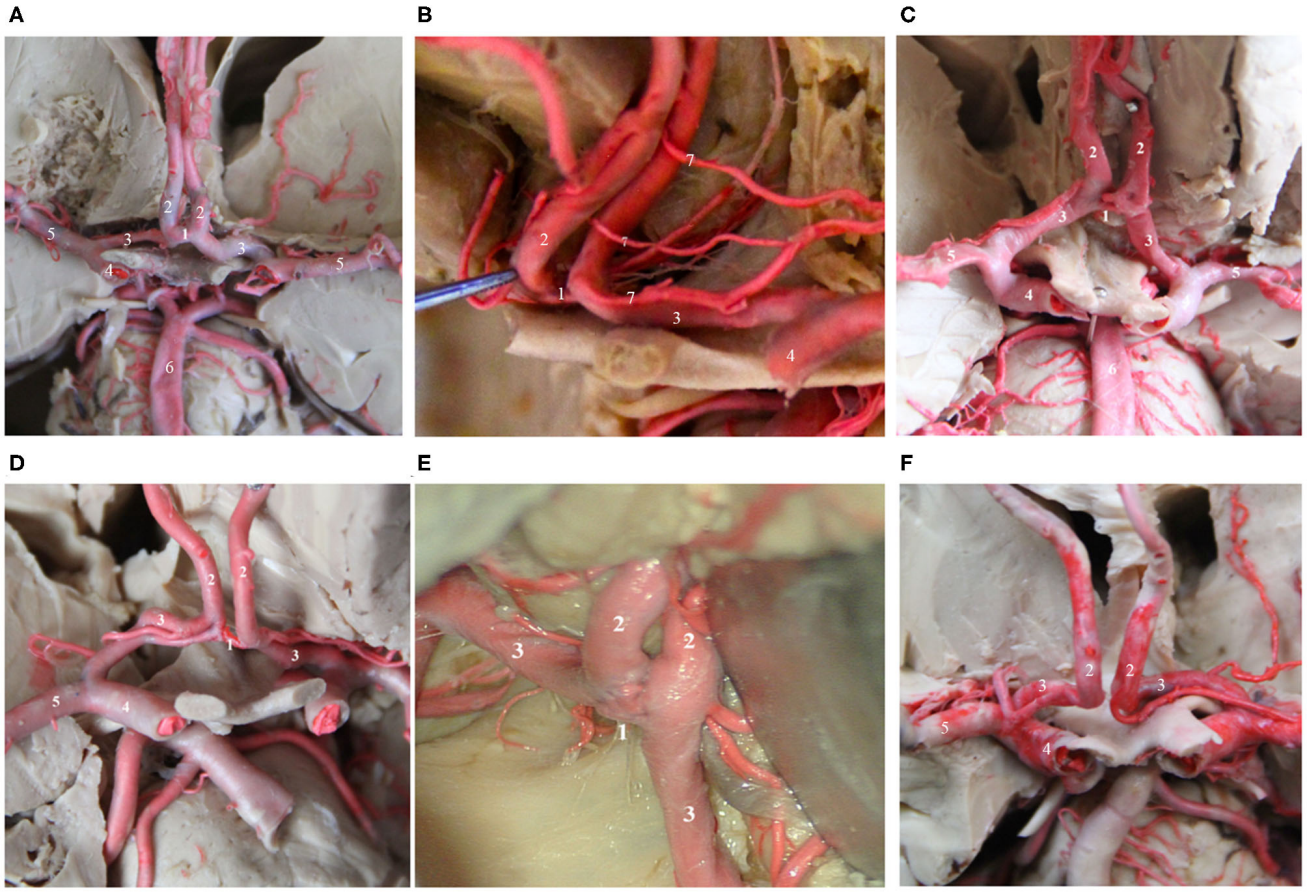
Several common AcoAs. **(A,B)**. Right A1 hypoplasia; **(C)** Duplicated AcoAs; **(D)** Right A1 segment fenestration; **(E)** AcoA fenestration deformity; **(F)** Absence of the AcoA. (1) AcoA; (2) A2 segment of the ACA; (3) A1 segment of the ACA; (4) MCA; (5) ICA; (6) Basilar artery; (7) recurrent artery of Heubner.

## Relationship Between AcoA and the A1 Segment of the Anterior Cerebral Artery (Figure 2)

The anterior cerebral artery is under the anterior perforated substance. After arising from the bifurcation of the internal carotid artery, it runs anteriorly, medially, and obliquely above the basal surface of the frontal lobe and the optic chiasm and connects with the contralateral anterior cerebral artery utilizing the AcoA in the longitudinal fissure. The A1 segment of the anterior cerebral artery exits the internal carotid artery cistern and enters the lamina terminalis cistern; the course is not straight and often shows various forms of bending, and therefore, attention should be paid to the course of the blood vessel when searching for the A1 segment of the anterior cerebral artery during clipping surgery. A study by Rhoton (19) showed that the diameter of the AcoA was usually 1 mm smaller than that of A1 on the dominant side, that 44% of the AcoA had a diameter ≤ 1.5 mm, and that 16% of the AcoA had a diameter ≤ 1 mm. The difference between A1 on the dominant side and A1 on the inferior side was related to the diameter of the AcoA (50% of the diameters of the A1 segments on the 2 sides were ≤ 0.5 mm); the mean diameter of the AcoA was 1.2 mm. Twelve percent of the diameter difference between A1 segments on the 2 sides were ≥1 mm; the mean diameter of the AcoA increased to 2.5 mm, which could be used for clinical estimation. Findings by Brandt (20) showed that ~21.05% of individuals had variations in the A1 segment of the anterior cerebral artery, such as a very large A1 segment on one side, while the contralateral A1 segment was very small or absent; this anatomical structure presents a high risk for the development of an AcoA aneurysm. This finding coincides with Rhoton's (19) suggestion that the greater the diameter difference between bilateral A1 segments, the more likely an AcoA aneurysm will develop. Yasargil (19) and Pandey and Steinberg (21) also reported that only 22% of patients presented comparable diameters of A1 segments of the bilateral anterior cerebral arteries, while unequal diameters of the bilateral A1 segments was significantly associated with the occurrence of AcoA aneurysms, and the aneurysm was usually located at the junction of the thicker A1 segment of the anterior cerebral artery and the AcoA. Paying attention to this anatomical characteristic and the hemodynamic feature is helpful for preoperative diagnosis and the selection of the surgical side and surgical approach (22, 23).

**Figure 2:**
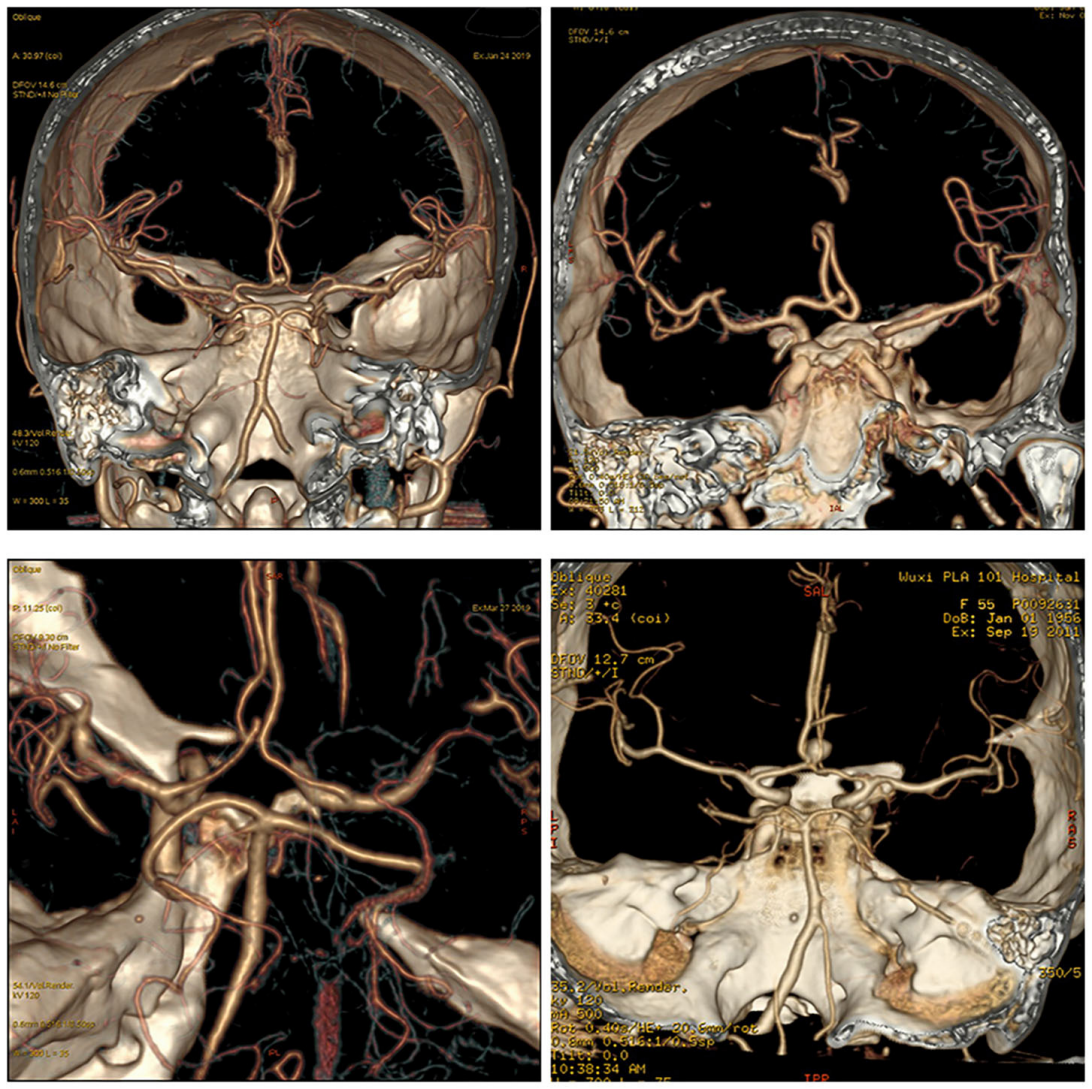
The relationship between the AcoA and bilateral A1. Different cases showed different relationships and locations for AcoA and bilateral A1.

## Recurrent Artery of Heubner (Figure 3)

In 1872, Heubner first described a recurrent artery that arises from the anterior cerebral artery and returns to the anterior perforated substance along the anterior cerebral artery. It is also the largest and most important perforating artery when the anterior cerebral artery is in the horizontal segment and is known as the medial striate artery or anterior striate artery. Its course is quite complex, and it is closely related to the anterior cerebral artery and the AcoA. The areas that receive blood supply from the recurrent artery of Heubner mainly include the thalamus, anterior lenticular nucleus, lateral globus pallidus, anterior caudate nucleus, anterior crus of the internal capsule and other important parts, and the recurrent artery of Heubner is the only perforating vessel at the base of the brain that supplies both the deep basal ganglia region of the brain and the cerebral cortex, such as the optic tract and the medial temporal cortex; its function is very important and must be taken seriously and protected during aneurysm clipping or aneurysm embolization (14, 24). Damage to this artery during surgery can lead to mild upper neurons paralyzes, such as muscle weakness of the contralateral tongue muscle and upper limb, and severe upper neuron paralysis of the contralateral lower limb. If the recurrent artery of Heubner on the dominant side is injured, symptoms such as aphasia can occur. Therefore, it is very important to identify the recurrent artery of Heubner and its origin and course before surgery. Almost everyone has bilateral Heubner's recurrent arteries, some of which have multiple branches, but very few studies have reported that there may be an absence of the recurrent artery of Gomes et al. (25) and González-Llanos et al. (26). The recurrent artery of Heubner originates from the medial segment of A1 of the anterior cerebral artery above the optic nerve and is adjacent to the AcoA. The diameter of the recurrent artery of Heubner is generally <2 mm, and most of these arteries have a bilaterally symmetrical structure (25). The course of the recurrent artery of Heubner is adjacent to the A1 segment of the anterior cerebral artery, the middle cerebral artery, and the internal carotid artery, it travels medially and superiorly to A1 and enters the important nucleus region of basal ganglia from the anterior perforated substance, and its terminal segment emits 1–4 branches that enter the brain (24). Protrusion-type AcoA aneurysms are more likely to be close to the recurrent artery of Heubner, and injury should be avoided when performing aneurysm clipping.

**Figure 3:**
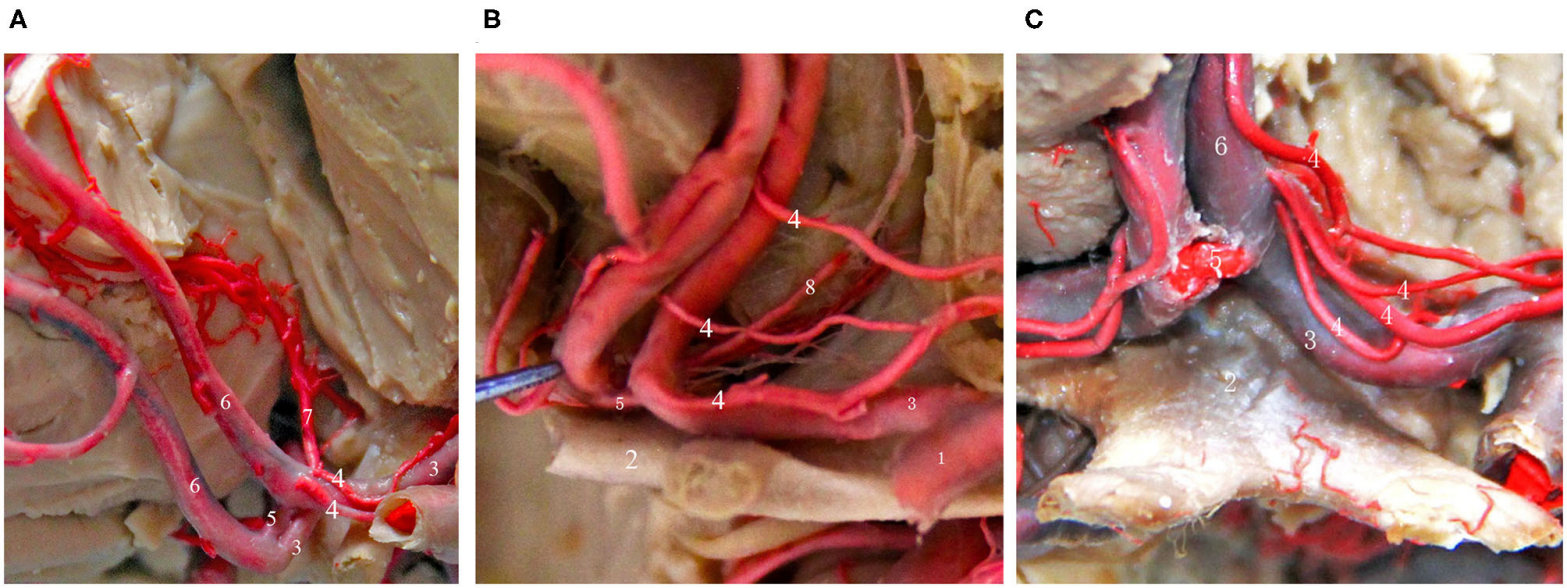
Common types of the recurrent artery of Heubner. **(A)** 2-branch recurrent artery (4 in Figure); **(B)** 3-branch recurrent artery (4 in Figure); **(C)** 4-branch recurrent artery (4 in Figure). (1) ICA; (2) Optic chiasma; (3) A1 segment of the ACA; (4) recurrent artery of Heubner. (5) AcoA; (6) A2 segment of the ACA; (7) Median callosal artery; (8) Frontopolar artery.

## Relationship Between the AcoA and Lamina Terminalis and Optic Chiasm

The lamina terminalis cistern is located in front of the lamina terminalis and extends forward into the longitudinal fissure. Its upper boundary is the anterior perforated substance, its lower boundary is the optic chiasm, its lateral boundary is the anterior cerebral artery, and its anterior wall is the posterior part of the bilateral straight gyrus. It is adjacent to the longitudinal fissure and bilateral olfactory cistern. The 2 sides of the cistern of lamina terminalis are adjacent to the proximal end of the Sylvian cistern and the internal carotid cistern or the internal carotid-posterior communicating artery cistern via the arachnoid of the anterior cerebral artery. Important blood vessels, such as the AcoA, the proximal segment of the A1 and A2 segments of the anterior cerebral artery, the recurrent artery, the perforating artery of the hypothalamus, and the anterior cerebral vein pass through the cistern of lamina terminalis. Through anatomical studies, Serizawa et al. (14) found that in 30% of people, the AcoA covers the lower third of the lamina terminalis; this was especially the case for an AcoA with plexiform or compound growth. In this case, if aneurysm clipping is performed, the trans-lamina terminalis surgical approach should not be used because the AcoA is susceptible to injury when the lamina terminalis is incised. The average distance between the midpoint of the posterior edge of the AcoA and the lamina terminalis is 11.8 ± 3.4 mm, the height to the superior surface of the optic chiasm is 2.1 ± 1.9 mm, and it is connected to the optic chiasm and the lamina terminalis by a short fiber trabecula in a roughly sagittal position (19). In the event of intraventricular hemorrhage due to the rupture of an AcoA aneurysm, surgical fenestration of the lamina terminalis can release cerebrospinal fluid and thereby release cerebral pressure. Superior and medial to the AcoA aneurysm is the gyrus rectus, which can be safely removed for better exposure. Adjacent to the gyrus rectus is the inferior frontal gyrus, which is associated with canonical anterior communicating aneurysmal amnesia, confabulation, and personality changes when injured. Due to the advancement of craniotomy and endovascular interventional treatment techniques, AcoA syndrome is rare. The optic chiasm is located below the AcoA, usually directly below it, but some are in an oblique position at an angle. Among them, the branches of the optic chiasm arising below the AcoA are distributed anterior to the optic chiasm and dorsal to the optic nerve and supply blood to the optic nerve and the superior part of the optic chiasm, but they are not the main blood supply arteries for this site because these vessels are thin. Some studies suggested that the main blood supply arteries for the optic chiasm and optic nerve come from a group of branches of the anterior cerebral artery (19, 27). In clinical practice, when rupture and bleeding of advanced AcoA aneurysms or other intracranial aneurysms are encountered, it is also possible to increase the retrochiasmatic space and release bloody cerebrospinal fluid to reduce intracranial pressure and relieve vasospasms by incising the lamina terminalis. If the aneurysm is found to be directed to or in contact with the lamina terminalis during AcoA aneurysm clipping, when blocking the A1 segment of the bilateral anterior cerebral arteries, the aneurysm body should be separated from the surrounding adhesions and flipped anteriorly and superiorly to facilitate clipping of the aneurysmal neck and avoid inadvertent injury to the perforating arteries.

## Relationship Between AcoA and Perforating Branch Vessels (Arising From the Posterior, Superior, Inferior Walls) (Figure 4)

Professor Serizawa et al. (14) conducted a careful study of the perforating vessels of the AcoA and divided them into 3 categories according to the specific supply sites of the perforating vessels. They suggested that all AcoAs had perforating vessels and that almost all of these perforating branches originate from the posterior, superior, and inferior walls of the AcoA. Through a large number of autopsies, some scholars have found that the perforating branches of the AcoA are almost always present and provide blood to the blood vessels of very important functional areas, such as the intracranial hypothalamus and optic chiasm. The appearance of sequelae, such as personality changes, disturbance of consciousness, mutism, and even internal environment disorder, after the surgical clipping or embolization of an AcoA aneurysm, is primarily due to the intraoperative damage to certain perforating branches of the AcoA (13). Because AcoA aneurysms are common intracranial aneurysms, it is very important to understand the anatomical structure and the variations in its perforating branches as well as the relevant preoperative images, regardless of surgical clipping or interventional embolization. Below, we briefly describe the specific classification, anatomical structure, and function of the perforating arteries of the AcoA.

**Figure 4:**
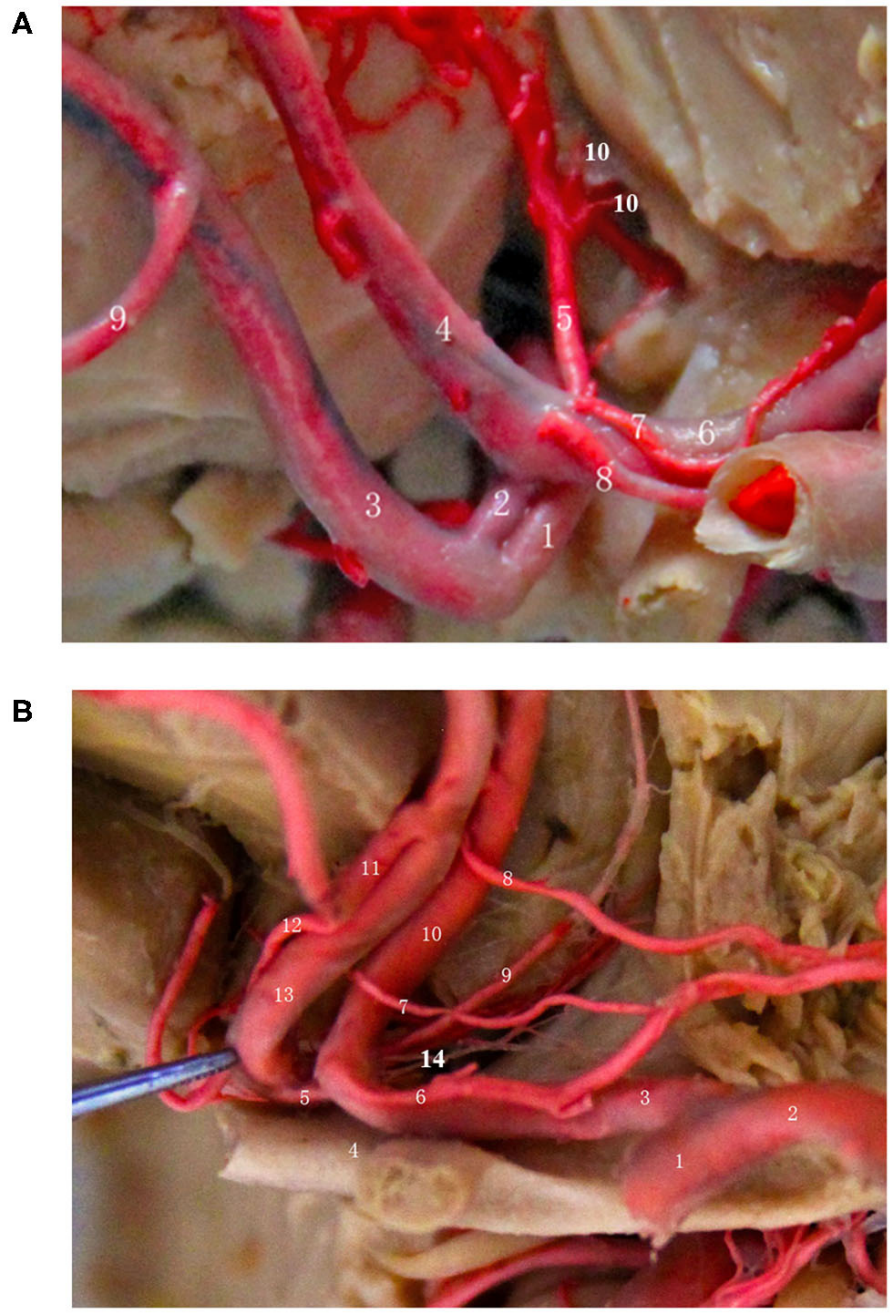
Relationship between the AcoA and perforating branch vessels (start from the posterior, superior, and inferior wall). **(A)** (1) Right A1 segment; (2) AcoA; (3) Right A2 segment; (4) Left A2 segment; (5) Median callosal artery; (6) Left A1 segment; (7/8) Left recurrent artery; (9) Frontopolar artery; (10) Perforating artery of the hypothalamus. **(B)** (1) Left internal carotid artery; (2) Left middle cerebral artery; (3) Left A1 segment; (4) Optic chiasm; (5) AcoA; (6/7/8) Left 3 recurrent arteries; (9) Subcallosal artery; (10) Left A2 segment; (11) Right medial frontobasal artery; (12) Right recurrent artery; (13) Right A2 segment; (14) Hypothalamic branches.

## Subcallosal Branch

The subcallosal branch, which is often large, is the thickest and most important perforating vessel of the AcoA, and sometimes it appears as a group of blood vessels formed by 2–4 branches. The probability of the existence of the subcallosal branch is ~13.7%, and its diameter is between 0.6 and 2.8 mm (28). The subcallosal branch arises from the posterosuperior region of the AcoA, runs toward the genu of the corpus callosum, and provides important branches to the hypothalamic area. Besides, it provides blood supply to areas including the lamina terminalis, anterior hypothalamus, anterior cingulate gyrus, rostrum and genu of the corpus callosum, anterior commissure, gyrus paraterminalis, fornix column, and septum pellucidum (14, 19). Besides, Serizawa et al. (14) was the first to distinguish the subcallosal artery from the median callosal artery. He suggested that the probability of the existence of the subcallosal artery was significantly higher than that of the median callosal artery and that they generally do not exist at the same time. They may come from the same vascular source, and the subcallosal artery may be an extension of the median callosal artery.

## Hypothalamic Branches

Most of the hypothalamic branches originate from the posteroinferior region of the AcoA and often coincide with the subcallosal branch. In general, the perforating arteries of the hypothalamus are a group of arteries of 2–6 branches that provide blood supply to the areas of the hypothalamus and lamina terminalis, and 10% originate from one side of the AcoA and end on the opposite side of the hypothalamic area. Symptoms such as water and electrolyte disturbances, disturbance of consciousness, cognitive impairment and high fever may occur if the perforating arteries of the hypothalamus are injured during surgery, and in severe cases, this type of injury is life-threatening; therefore, it is critically important to carefully identify and protect these arteries during surgery (29). It is not necessary to cut the AcoA to increase the surgical exposure space or because of its large size. Once the AcoA is cut off, it is very easy to accidentally injure the perforating artery of the hypothalamus and thus cause symptoms of hypothalamic injury. It is particularly important to protect the hypothalamus and its perforating artery when the frontal longitudinal fissure approach is used to remove space-occupying lesions from the sellar region.

## Optic Chiasm Branch

The chiasmatic perforating artery has the lowest occurrence frequency among the 3 blood vessels, and it has been reported that the probability of its occurrence is ~10%. It usually originates from the posteroinferior region of the AcoA, has a diameter of 0.1–0.2 mm, and provides blood supply to the optic nerve and the region above the chiasm, but it is not a major artery for blood supply to this region. This site receives blood supply from both the superior hypophyseal artery and the chiasmatic artery and has abundant anastomotic branches; therefore, if necessary, cutting off the small perforating arteries that connect the AcoA and the optic chiasm generally do not cause severe symptoms after surgery. However, patients with ruptured AcoA aneurysms often have vasospasms. If the chiasmatic artery and its perforators are severed in the presence of superior hypophyseal artery spasms, there is a possibility of decreased visual acuity and visual field defects.

## Anatomic Characteristics of AcoA Aneurysms and Their Relationship With Surgery

The anatomical characteristics of an AcoA aneurysm determine whether microsurgery or interventional embolization treatment is preferred, the size and direction of the aneurysm determine the difficulty level of the surgery and the choice of the optimal surgical approach, and whether the aneurysm is accompanied by calcification and intra-aneurysm thrombosis determines the precautions during surgical clipping and possible complications (22, 23). At present, no literature has summarized the relationship between the anatomical characteristics of AcoA aneurysms and surgery. Also, there is no unified understanding of the side selection for the surgical treatment of AcoA aneurysms. Traditionally, the non-dominant hemisphere side is generally selected for craniotomy. Therefore, mastering the anatomical characteristics of the aneurysm itself and knowing the preoperative imaging results are helpful to the successful completion of the surgery and the reduction in postoperative complications. In the following, we briefly describe the relationship between the anatomical characteristics of aneurysms and surgery according to the structural characteristics of aneurysms and the latest literature.

## Relationship Between Aneurysm Size and Surgery

The size of an AcoA aneurysm is a crucial factor affecting treatment decisions, such as whether the aneurysm requires surgical intervention, whether surgery or intervention treatment is the preferred choice, and the specific treatment strategies. The ISUIA reports that smaller intracranial aneurysms in the anterior circulation are associated with a lower risk of spontaneous rupture, and the annual risk of rupture of anterior circulation aneurysms with a diameter <7 mm is <1% (6); therefore, the need to treat unruptured AcoA aneurysms with a diameter <7 mm remains highly controversial. However, increasingly more studies have reported that smaller diameter AcoA aneurysms are associated with a high risk of rupture. Bijlenga et al. (9) found that the risk of rupture risk for more than 900 AcoA aneurysms with a size of 4–7 mm was similar to that for posterior circulation aneurysms. In a study of 200 ruptured aneurysms, Lee et al. (10) found that 47% were small aneurysms (<5 mm) and that the most common rupture site was at the AcoA. Matsukawa et al. (4) found that AcoA aneurysm rupture was more likely to occur in younger patients (<60 years) and patients with combined high cholesterol and an aneurysm with forwarding protrusion and a daughter sac. Yamamoto et al. (30) reported 104 ruptured AcoA aneurysms with a mean diameter of 5.0 ± 1.8 mm. Through a meta-analysis of the literature, Steklacova et al. (31) found that ruptured AcoA aneurysms with a diameter <7 mm accounted for 67% of ruptures. Therefore, whether an AcoA aneurysm requires surgery cannot be determined solely based on whether the diameter of the anterior circulation aneurysm is <7 mm. It is generally recommended that for unruptured AcoA aneurysms with a diameter >3 mm, aggressive surgical treatment may be indicated to reduce the incidence of hemorrhage, especially for patients with a history of subarachnoid hemorrhage, an irregular-shaped aneurysm, imaging evidence of progressive growth, and combined hypertension and a history of smoking.

The size of an AcoA aneurysm not only affects the surgical indication but also plays an important role in the choice of surgical procedure. Numerous studies have found that if an AcoA aneurysm is too small or the aspect ratio (dome/neck > 1:2) is not appropriate, interventional embolization is usually not appropriate. Surgical clipping is preferred for small AcoA aneurysms because interventional embolization is limited by the coil size and the difficulty of placing a microwire or a microcatheter into the location (11, 32, 33). Some studies have even reported that in some AcoA aneurysm embolization procedures, the surgical procedure has been changed to surgical clipping due to finding a small aneurysm diameter or an inappropriate aspect ratio (dome/neck) (11). With the development of new balloons, stents, and more optimized catheter techniques, small or tiny AcoA aneurysms can also be successfully treated by interventional embolization; however, at present, it is not widely used, and long-term follow-up data are lacking (34–38). Some studies have investigated the relationship between the size of an AcoA aneurysm and the risk of intraoperative rupture, especially during interventional embolization. Among them, Sluzewski et al. (34) reported 264 cases of aneurysm embolization. There were 7 cases of intraoperative rupture, including 4 cases of ruptured aneurysms with a diameter <4 mm; however, none of the 4 cases was an AcoA aneurysm. Nguyen et al. (35) reported that during the embolization of aneurysms with a diameter <3 mm, the risk of aneurysm rupture was 11.7 and 35% of the ruptured microaneurysms were located in the AcoA. For the first time, professor Schuette et al. (36) conducted a cohort study of 347 patients with AcoA aneurysms, followed them continuously for 10 years, and found that the risk of rupture during aneurysm embolization was significantly increased when the diameter of the AcoA aneurysm was <4 mm (compared with the risk of rupture during embolization of AcoA aneurysms with a diameter > 4 mm, 13.5% vs. 2.9%) and that aneurysm rupture affects the final prognosis of patients.

## Relationship Between Aneurysm Direction and Surgery

The direction of an AcoA aneurysm is an important factor affecting the choice of the side of surgical clipping, while different sides could affect the degree of difficulty in exposing the aneurysm neck and bilateral A1 and A2, which may also affect the efficacy of surgical clipping (18). According to a report by Suzuki et al. (22), based on a DSA or CTA lateral view, the coordinates were established with the aneurysm as the base point and the parallel line of the sphenoid platform as the transverse axis, and the longitudinal axis was perpendicular to the transverse axis. AcoA aneurysms are classified into anterosuperior, anteroinferior, posterosuperior, and posteroinferior depending on the direction of the aneurysm, and superiorly projecting AcoA aneurysms are the most common, accounting for 19–37% (23, 39, 40). Superiorly projecting AcoA aneurysms are often located in the deep portion of both hemispheres. The main body and the neck of aneurysms can be easily blocked by the junction of the A1-A2 segments of the AcoA as well as the closed A2 segment, and it is difficult to separate the aneurysm and expose the contralateral A2 segment during surgery. To expose the aneurysm, it may be necessary to resect the gyrus rectus; therefore, the difficulty of the operation and postoperative complications are greatly increased. For posteriorly projecting aneurysms, the recurrent artery and the contralateral A1 segment can be injured while exposing the aneurysm. Aneurysms with different projections have different hemodynamics, causing shear forces on the aneurysm wall, pressure of blood flow on the aneurysm wall, and intra-aneurysm blood flow velocity to be different; therefore, the risk of rupture and the growth rate are different for aneurysms pointing different directions (40–45). Shao et al. (44) found that the risk of rupture was significantly increased in anteriorly projecting AcoA aneurysms compared with that for posteriorly projecting aneurysms, and this finding was later confirmed by extensive literature, suggesting that anteriorly projecting AcoA aneurysms are more likely to rupture (4, 43) and that the Fisher grade after bleeding is high and the prognosis is relatively worse (18, 40). Therefore, posteriorly projecting AcoA aneurysms have a low risk of rupture.

For side selection in the microsurgical clipping of AcoA aneurysms, the traditional view suggests choosing the non-dominant hemisphere for craniotomy (usually the right side), unless the dominant side is associated with multiple aneurysms of intracranial hematomas (39). Also, some scholars suggest the A1 dominant blood supply side as the surgical approach to facilitate the immediate control of blood supply to the aneurysm and exposure of the aneurysm neck (40). Through a large sample study, Bohnstedt et al. (40) found that only 58.6% of patients achieved an acceptable prognosis when craniotomy and AcoA aneurysm clipping were carried out from the A1 dominant side, while 100% of the patients achieved an acceptable prognosis when craniotomy and AcoA aneurysm clipping were carried out from the contralateral side of the A1 dominant side; he suggested that this may be related to the anteroposterior locations of the bilateral A2 segment of anteriorly projecting AcoA aneurysms. A study by LeRoux et al. (46) suggested that the reason for choosing the dominant hemisphere for craniotomy maybe because the A1 on the non-dominant hemisphere side could not be detected by imaging or because the aneurysm arose from the intersection of the AcoA and A2 on the dominant hemisphere side.

**Figure 5:**
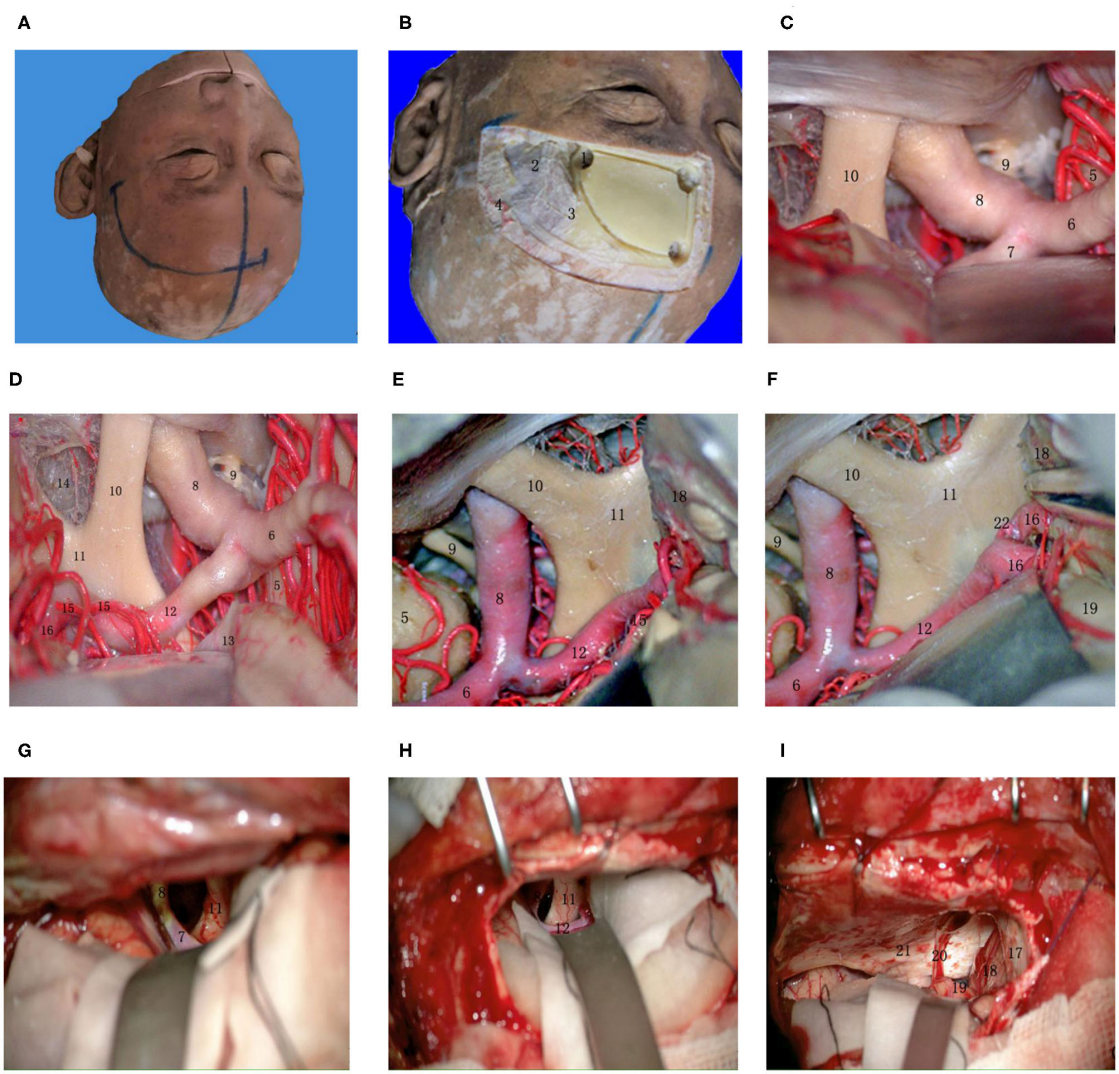
Supraorbital lateral-longitudinal fissure approach to the AcoA. **(A)** Midline and scalp incision; **(B)** To identify key holes and identify bone windows; **(C)** Exposing the beginning of the A1 segment of the anterior cerebral artery at the lateral orbit; **(D)** Exposing the whole process of the A1 segment of the anterior cerebral artery; **(E)** Separating the longitudinal fissure and exposing the A1 segment of the anterior cerebral artery; **(F)** Exposing the anterior communicating artery complex; **(G)** Exposing the beginning of the A1 segment of the anterior cerebral artery at the lateral orbit during a real surgery; **(H)** Exposing the whole process of the A1 segment of the anterior cerebral artery during a real surgery; **(I)** Raising the frontal lobe next to the midline, exposing and separating the olfactory bundle. (1) key hole; (2) temporalis muscle; (3) superior temporal line; (4) superficial temporal artery; (5) temporal lobe; (6) MCA; (7) beginning of the A1 segment of the ACA; (8) ICA; (9) oculomotor nerve; (10) optic nerve; (11) optic chiasma; (12) A1 segment of the ACA; (13) base of the frontal lobe; (14) anterior cistern of the optic chiasma; (15) recurrent artery; (16) A2 segment of the AcoA; (17) cerebral falx; (18) medial frontal lobe; (19) gyri rectus; (20) olfactory bundle; (21) anterior cranial fossa; (22) AcoA.

**Figure 6:**
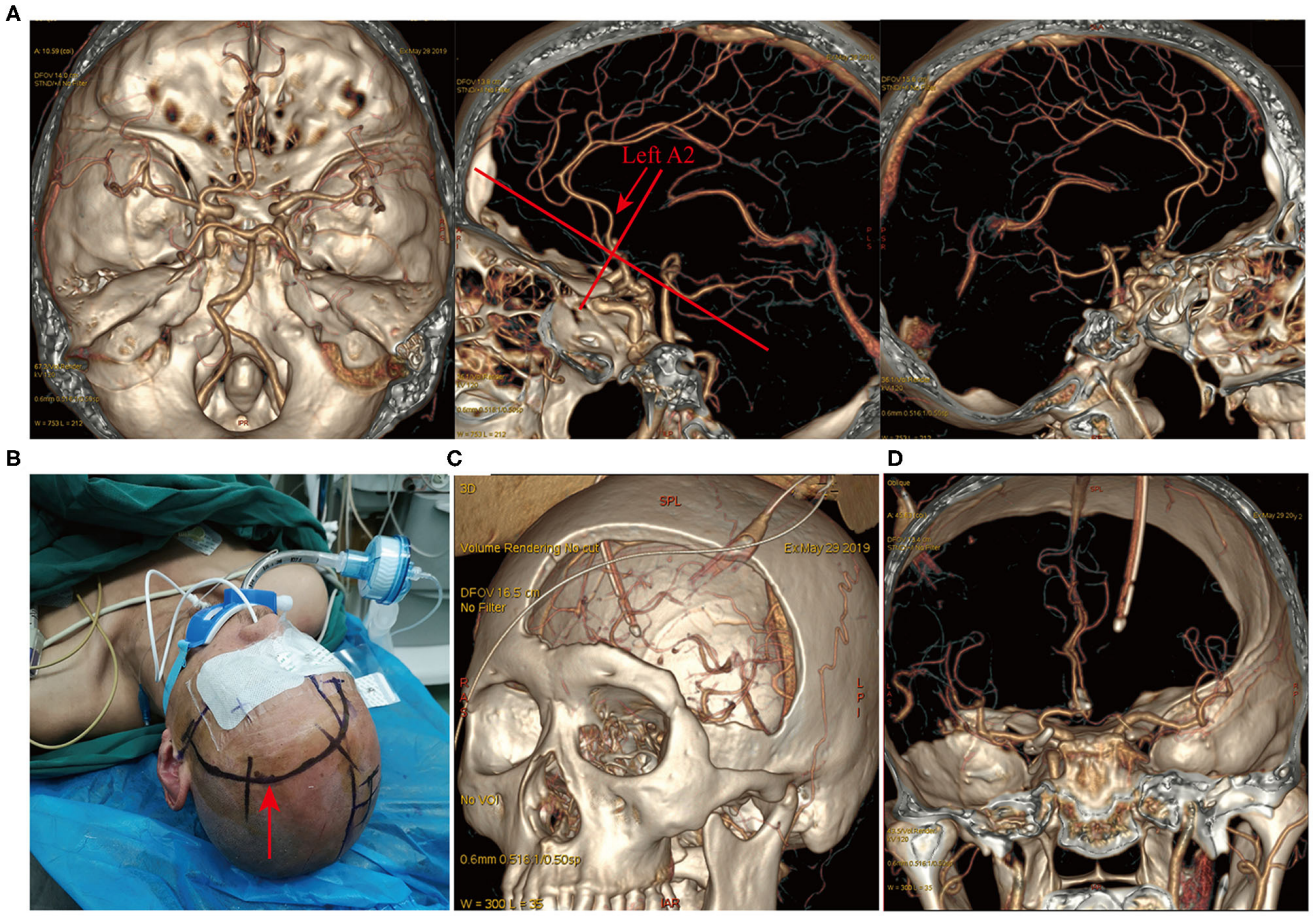
Longitudinal fissure pterion surgical approach. A patient with ruptured multiple intracranial aneurysms and hemorrhage (Hunt-Hess grade IV). **(A)** CTA revealed a left posterior communicating artery aneurysm and an AcoA aneurysm. Lateral view showed a posteriorly projecting AcoA aneurysm and the left A2 posterior to the right A2 (indicated by the red arrow). The left longitudinal fissure pterional approach was selected for surgery. **(B)** Surgical incision and patient positioning. **(C)** Postoperative three-dimensional CT reconstruction indicated the size and location of the surgical bone window. **(D)** Postoperative CTA revealed complete clipping of the AcoA aneurysm and the left posterior communicating artery aneurysm, with no remnants and good vessel protection.

The direction of an AcoA aneurysm not only affects the choice of the surgical side but also determines the choice of surgical approach as well as the difficulty of the operation. We know that AcoA aneurysms can almost always be resolved by the conventional pterional approach, but different surgical approaches determine the difficulty of the surgery and the resection rate of the gyrus rectus. Depending on the severity of the subarachnoid hemorrhage, the size and direction of the aneurysm, the supraorbital, longitudinal fissure, pterion, and orbitozygomatic approaches are good choices. Through autopsy and numerous clinical studies, we have found that the supraorbital and longitudinal fissure approach can better expose the AcoA complex, especially the superior and posterosuperior regions of the AcoA, and the traction on the ipsilateral frontal lobe and gyrus rectus is significantly reduced at the time of exposure. This approach is very helpful for exposure and clipping of superiorly and posteriorly projecting AcoA aneurysms as well as complex AcoA aneurysms (Figure 5). ② The trans-longitudinal fissure pterional approach has a good operative field (Figure 6), less traction on the brain tissue, and less vascular damage. It allows clipping the aneurysm at various angles from the anterior longitudinal fissure to the pterion, allowing intraoperative adjustment of the aneurysm clip according to aneurysm size and direction; furthermore, it is more valuable for complex AcoA aneurysms or posteriorly projecting aneurysms. The above 2 surgical approaches are more beneficial for clipping superiorly and posteriorly projecting AcoA aneurysms. Because of the relatively large bone window, it is more suitable for patients with advanced conditions or with relatively severe intracranial conditions or with more subarachnoid hemorrhage and intracranial hematoma. The longitudinal fissure approach can be used to incise the corpus callosum and then remove the intraventricular hematoma, which facilitates cerebrospinal fluid circulation.

## Relationship Between Aneurysm Morphology and Surgery

At present, the detection rate of unruptured intracranial aneurysms is increasing due to the promotion of non-invasive CTA technology and the improvement in people's awareness of cerebrovascular diseases. Therefore, the evaluation of the risk of rupture of unruptured aneurysms has also become a research hotspot. Numerous relevant studies have reported the relationship between AcoA aneurysm morphology and the risk of rupture (4, 35, 43, 47–50), but most have not conducted a systematic study, and there is a lack of analysis of the relationship between aneurysm morphology and surgery (43). Although there are many characteristics related to the morphology of an AcoA aneurysm, the characteristics that have the most predictive value for the risk of rupture may be whether the aneurysm has a fenestration deformity, the direction of the aneurysm body, and the ratio (size ratio) of the aneurysm height (longest distance from the aneurysm body to the midpoint of the neck) to the mean diameter of the adjacent 4 blood vessels [(sum of the diameters of bilateral A1 and A2)/4] (43). The aneurysm direction has been well-documented to be closely related to aneurysm rupture, which has been reviewed previously (4, 11, 40, 41, 44, 45, 47–50). The presence or absence of fenestration deformity is a very important feature that can be used to predict the risk of rupture. If an aneurysm is found to be associated with fenestration deformity on CTA/DSA, its risk of rupture is significantly increased or the risk of re-rupture is significantly increased (50, 51). By studying the characteristics of 255 AcoA aneurysms, Choi et al. (50) found that the association of AcoA aneurysms with fenestration deformity was an independent risk factor for predicting rupture. Additionally, de Gast et al. (51) also reported that the fenestration deformity of the AcoA was associated with aneurysm development. The reason may be related to the fenestration deformity caused by the incomplete development and maturation of the arterial wall and the turbulence of blood flow caused by the lack of an elastic middle layer of the arterial wall (52). Another most important factor is the size ratio; the diameter of the bilateral A1 and A2 segments adjacent to the aneurysm may affect the hemodynamics of the aneurysm. The larger the size ratio is, the more complex the hemodynamics of the aneurysm. In the presence of multiple turbulent flows and low tangential stress on the aneurysm wall, the risk of aneurysm rupture is high (53, 54). Therefore, the size ratio is an independent risk factor for AcoA aneurysm rupture (43, 53, 54). Besides, the relationships between aneurysm size, aneurysm angle, and aneurysm location, and the rupture risk have not been reviewed because there are few reports or there are considerable controversies (43). Because aneurysm morphology has an important influence on aneurysm rupture, treatments should be carefully selected when unruptured aneurysms are encountered in clinical practice. For ruptured aneurysms, if the size ratio is large or if the aneurysm body is superiorly projecting or has fenestration deformity, it should be treated aggressively at an early stage to prevent aneurysm re-rupture.

Interventional embolization and craniotomy and clipping are both treatments for AcoA aneurysms; however, both treatments are complex and difficult relative to other treatments for intracranial aneurysms. Whether it is easier to use aneurysm embolization or clipping depends on the shape and size of the aneurysm and the relationship with adjacent blood vessels. Therefore, a preoperative assessment of aneurysm morphology is very important. Embolization of large or wide-necked aneurysms can be difficult (34, 55–57) and requires assistance with stents or balloons, which increases the procedure-related disability and mortality rates (58, 59). The development of new interventional materials and improvements in interventional embolization techniques have led to the safe embolization of an increasing number of AcoA aneurysms that were previously difficult to treat by interventional embolization or at great risk of embolization (60–66). In particular, the application of the latest flow diverters has made treatment simple and safe for many intracranial wide-necked or microvascular aneurysms and recurrent complex aneurysms that were previously difficult to treat by simple embolization (61, 62, 64–66). Besides, some new special devices, such as the pCONUS (phenox GmbH, Bochum, Germany) stent (63) and the LVIS Junior (LVIS Jr. MicroVention, Aliso Viejo, CA, USA) stent (60), maybe safe and more effective for the treatment of wide-necked or complex AcoA aneurysms. Taken together, for selecting a suitable and safe treatment for AcoA aneurysms, in addition to the above relevant anatomical factors, each hospital should take specific considerations according to its specific circumstances, the patient's selection and financial resources, the availability of the materials and instruments and the comprehensive capabilities of the facility.

## Influence of the Location Relationship Between the Aneurysm and Bilateral A1 and A2 on Surgery

Because the anatomical structure of bilateral A1 and A2 plays an important role in the formation of AcoA aneurysms, the diameters of bilateral A1 and A2 and their proportional relationship is closely related to the formation of aneurysms and the starting position of aneurysms, and the diameter ratio for A1 and A2 is closely related to the risk of aneurysm rupture (67). For clipping surgeries or interventional embolization of AcoA aneurysms, the diameters of bilateral A1 and A2, as well as their anatomical location relationship with the aneurysm, must be considered because they determine the side choice for surgical clipping, the difficulty of the operation and whether a stent is needed during embolization. Therefore, it is important to conduct a meta-analysis of the influence of the anatomy of bilateral A1 and A2 on AcoA aneurysm surgery according to the literature.

Numerous studies have found that patients with dominant side A1 blood supply have a higher probability of forming an AcoA aneurysm, which may be closely related to the hemodynamic profile caused by the imbalances resulting from inflow from the dominant side A1 and inflow from the inferior side A1 (39, 68, 69). Previous studies have suggested that the presence of a dominant side A1 and the dysplasia or deficiency of A1 on the contralateral side are key factors for the formation of AcoA aneurysms (68–71). Some scholars also studied the relationship between the diameter ratio of A1 and A2 and the formation and rupture of aneurysms and found that regardless of the presence of a dominant side A1, the smaller the diameter of A2 and the larger the diameter ratio of A1/A2, the higher the probability is of forming an AcoA aneurysm and the greater the risk of rupture (67). Some scholars also studied the relationship between the angle of the A1 and A2 segments and the risk of aneurysm formation and rupture. Ye et al. (71) compared CTA imaging data of 64 patients with AcoA aneurysms and 187 patients without AcoA aneurysms and found that the smaller the angle was between A1 and A2, the higher the probability was of developing an aneurysm; furthermore, they confirmed that the probability of developing an AcoA aneurysm was significantly higher in patients with a dominant side A1. These conclusions are almost the same as the findings by Kasuya et al. (72). In addition to studying the diameter and angle of A1 and A2, some studies have found a relationship between the absence or dysplasia of the A1 segment and aneurysms. Yang et al. (73) retrospectively analyzed the data of 251 patients with AcoA aneurysms and found that 49.8% of patients had hypoplasia of the A1 segment. Univariate analysis revealed that hypoplasia of the A1 segment was closely related to anterior cerebral artery infarct and prognosis after aneurysm clipping. Multivariate analysis revealed that hypoplasia of the A1 segment was also a strong independent risk factor for anterior cerebral artery infarct and poor prognosis after aneurysm clipping. Rinaldo et al. (74) also reported that hypoplasia of the A1 segment was an important factor in the formation of aneurysms.

**Figure 7:**
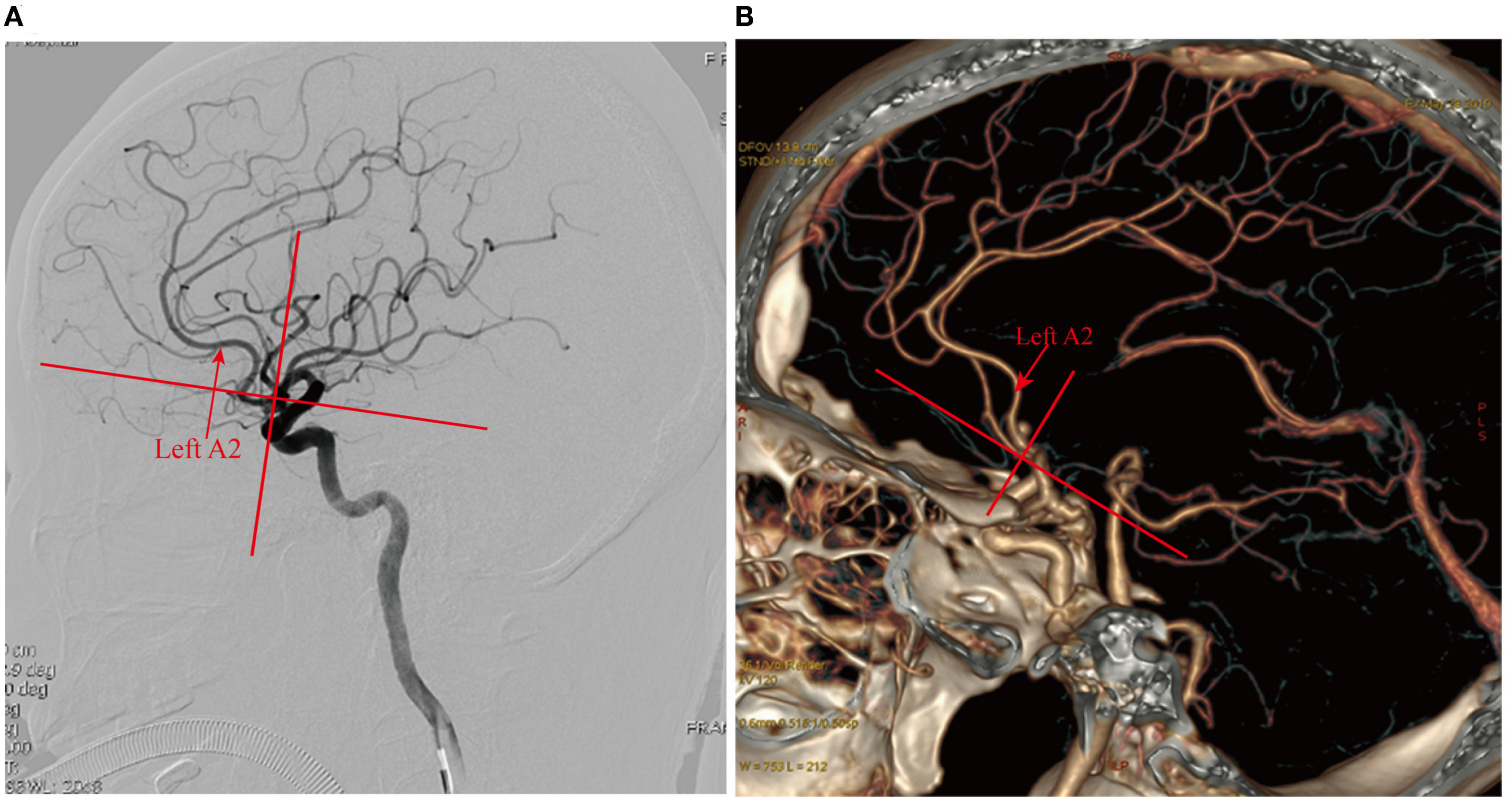
Selection of the operative side based on the anteroposterior relationship of the bilateral A2. **(A)** DSA after spontaneous subarachnoid hemorrhage showed superiorly projecting microanterior communicating aneurysms, the left A2 located anterior to the right A2, and the left side as the closed side; the right side should be selected as the operative side; **(B)** CTA examination after spontaneous subarachnoid hemorrhage showed a superiorly projecting AcoA aneurysm, the left A2 posterior to the right A2, and the left side as the open side; the left side should be selected as the operative side.

As we reviewed earlier, although almost all AcoA aneurysms can be clipped through the pterional approach in the non-dominant hemisphere, the choice of different sides, as well as the choice of different surgical approaches, may be related to the difficulty of the aneurysm clipping surgery, the resection rate of the gyrus rectus and postoperative complications (22, 23). Suzuki et al. (22) was the first to classify the relationship between bilateral A2 of superiorly projecting AcoA aneurysms with the open A2 plane side (when the pterional approach is used on this side, A2 on this side near the aneurysm body is located more posteriorly than the contralateral A2) and the closed A2 plane side (when the pterional approach is used on this side, A2 on this side near the aneurysm body is located more anteriorly than the contralateral A2). It is very difficult to expose the aneurysm neck during an operation on the closed A2 plane side, and it may be necessary to remove the gyrus rectus and pull the A2 segment during the operation, which may easily lead to a residual aneurysm neck and more postoperative complications. In subsequent studies, Hyun et al. (23) also proposed a relationship between bilateral A2 and concluded that surgery on the closed A2 plane side may be associated with more postoperative complications, and the use of the open A2 plane side for the operation may be easier and safer for exposing the aneurysm neck and may reduce postoperative complications. However, the number of cases in the above studies is small, and there is a lack of multicenter, randomized controlled studies to confirm the results. After summarizing the long-term experience of AcoA aneurysm clipping, our center concluded that according to CTA results and CTA surgical simulation images, for superiorly or anteriorly projecting AcoA aneurysms, the surgical approach should be determined according to whether the A2 on the pterional approach side is located more anteriorly or posteriorly than the contralateral A2, and it may be more beneficial for surgical clipping when the surgical approach side A2 is located more posteriorly than the contralateral A2 (Figure 7). We have submitted a multicenter randomized controlled trial registration to the Chinese Clinical Trial Registry to investigate clipping side choice for superiorly or anteriorly projecting AcoA aneurysms. In conclusion, side selection for AcoA aneurysm clipping should be individualized for the optimal surgical outcomes and minimal surgical injury based on imaging findings and 3D-CTA surgical approach simulation results.

## Summary

AcoA aneurysms are the most common intracranial ruptured or unruptured aneurysms, and surgical clipping or endovascular embolization of these aneurysms is a challenge faced by neurosurgeons. Because AcoA aneurysms have relatively complex anatomical structures, have anatomical variations, and are adjacent to important blood vessels and structures, it is necessary to pay attention to not only the anatomical characteristics of the aneurysm itself but also to the important adjacent blood vessels and perforating branches when exposing an aneurysm. Therefore, AcoA aneurysms are the most difficult intracranial aneurysm to resolve. With the continuous development of interventional techniques and the continuous improvement in interventional materials, most AcoA aneurysms can now be treated by endovascular techniques. Because current stent techniques include flow-diverting devices, even giant or thrombotic aneurysms can be effectively treated by endovascular methods. However, surgical clipping still retains an important method in some cases, especially for giant wide-necked aneurysms for which isolation and bypass may be the only optimal treatment. Also, the BRAT study showed no difference in risk and clinical outcomes between microsurgical clipping and coiling embolization, while the embolization group had higher recurrence and retreatment rates. For AcoA aneurysms with relatively complex structures, the probability of incomplete embolization and recurrence may be high. Therefore, microsurgical clipping is a good treatment for AcoA aneurysms.

The probability of variation in the AcoA and its perforating branches is very high, but they must be preserved as much as possible during operation, especially the subcallosal branches and the hypothalamic branches. Variation in the AcoA and the relationship between bilateral A1 and A2 are also important factors in the formation of aneurysms. Numerous studies have summarized the relationship between the AcoA and adjacent vessels and aneurysm formation. Factors such as fenestration deformity of the AcoA, size ratio, and the presence of A1 on the dominant side, have provided important reference for us to further study the formation of AcoA aneurysms. In terms of the choice of surgical and interventional treatments, treatments should be administered according to actual circumstances and should not be generalized because many factors affect the choice of treatment, such as the shape and size of the aneurysm, the anatomical relationship with the adjacent blood vessels, patient conditions, and the neurosurgeon's mastery of various treatment techniques.

For most AcoA aneurysms, it is possible to perform craniotomy through the pterion, and for aneurysms with good morphology, the supraorbital keyhole approach can also be used to achieve good clipping. For some aneurysms with special directions, combined approaches, such as the extra-orbital and lateral-longitudinal fissure approach or longitudinal fissure pterional approach, may have unique advantages, but the imaging characteristics need to be carefully analyzed before surgery. For the selection of the operative side, the conventional idea is to use the non-dominant hemisphere side and the A1 dominant blood supply side. However, for some aneurysms with special directions (superiorly projecting aneurysms), the anteroposterior relationship of bilateral A2 could affect the exposure of the aneurysm neck, the difficulty of operation, the resection rate of the gyrus rectus and the postoperative complications. Therefore, more research is needed to confirm the corresponding side selection, providing a very valuable idea for us to more elaborately study surgical strategies for AcoA aneurysm treatment.

## Author Contributions

JC, YW, and QC designed this research. XZ, CZ, WS, and YC collected the relevant literatures. JC, XZ, and ML wrote and revised the manuscript. YC revised the final manuscript critically for important intellectual content. YW and QC agreed to be accountable for all aspects of the work in ensuring that questions related to the accuracy or integrity of any part of the work are appropriately investigated and resolved. All authors have read and approved the final manuscript.

## Conflict of Interest

The authors declare that the research was conducted in the absence of any commercial or financial relationships that could be construed as a potential conflict of interest.

## Abbreviations

AcoA: Anterior communicating artery
CTA: computerized tomography angiography
DSA: Digital Substraction Angiography
ACA: Anterior cerebral artery.
