# Corrigendum: Anterior Communicating Artery Aneurysms: Anatomical Considerations and Microsurgical Strategies

**DOI:** 10.3389/fneur.2020.620226

**Published:** 2020-11-20

**Authors:** Junhui Chen, Mingchang Li, Xun Zhu, Yan Chen, Chunlei Zhang, Wenwen Shi, Qianxue Chen, Yuhai Wang

**Affiliations:** ^1^Department of Neurosurgery, Renmin Hospital of Wuhan University, Wuhan, China; ^2^Department of Neurosurgery, 904th Hospital of Joint Logistic Support Force of PLA, Wuxi Clinical College of Anhui Medical University, Wuxi, China; ^3^Department of Internal Medicine, Hexian Hospital of Traditional Chinese Medicine, Ma'anshan, China

**Keywords:** AcoA aneurysm, anatomical, microsurgical strategies, clipping, coiling, surgical strategy

In the published article, there was an error in the affiliation for authors Mingchang Li, Qianxue Chen. Instead of “Department of Neurosurgery, 904th Hospital of Joint Logistic Support Force of PLA, Wuxi Clinical College of Anhui Medical University, Wuxi, China,” it should be “Department of Neurosurgery, Renmin Hospital of Wuhan University, Wuhan, China.” There was an error in the affiliation for authors Xun Zhu, Chunlei Zhang, and Yuhai Wang. Instead of “Department of Neurosurgery, Renmin Hospital of Wuhan University, Wuhan, China,” it should be “Department of Neurosurgery, 904th Hospital of Joint Logistic Support Force of PLA, Wuxi Clinical College of Anhui Medical University, Wuxi, China.”

The authors apologize for this error and state that this does not change the scientific conclusions of the article in any way. The original article has been updated.

